# Supplementary material for: Thalamic input to the lateral amygdala determines the temporal window of fear-memory association
Source: Commun Biol. 2025 Jun 5;8:873. doi: 10.1038/s42003-025-08289-0 (PMC12141508; doi:10.1038/s42003-025-08289-0)

Supplementary Figures – “Thalamic input to the lateral amygdala determines the temporal window of fear-memory association”

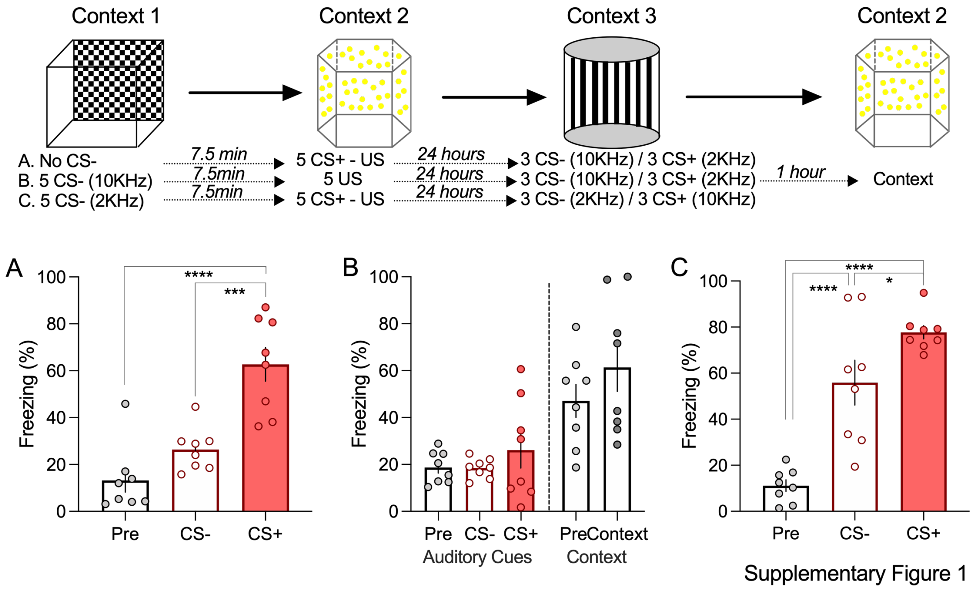

Supplementary Figure 1

Supplementary Figure 1. Memory association is established between the two auditory cues. A. If the CS- in not present during the first event, memory association is not observed and animals selectively respond to the CS+ (ANOVA F=22.64 P<0.0001; ****P<0.0001 ***P=0.0003 n=8). B. If the CS+ is not present during the second event, no association between CSs and US is established and animals do not respond to either cues in the test trial (ANOVA F (2, 21) = 0.86 P=0.4367). Animals do respond to the training context showing that the absence of response to cues is not due to a lack of learning. C. Switching auditory cues used as CS- and CS+ does not block memory association (F (2, 21) = 31.57 P<0.0001; * P=0.046 **** P<0.0001 **** P<0.0001). Error bars represent SEM.

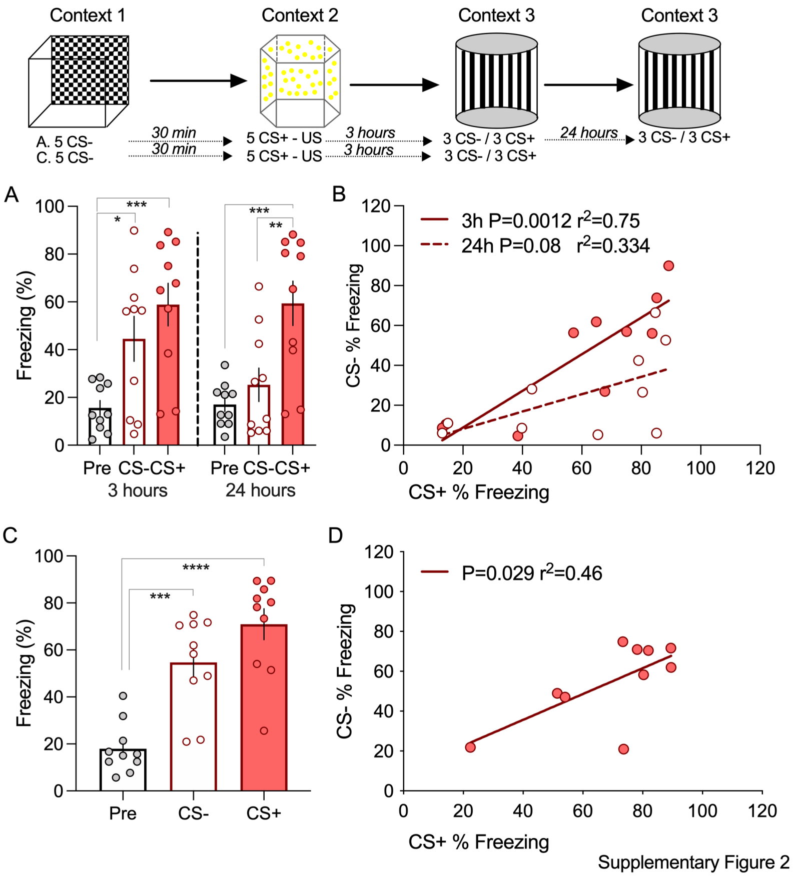

Supplementary Figure 2

Supplementary Figure 2. A. Exposing animals to two events with a 30 minutes interval leads to the formation of an associative fear memory that is already observable if animals are tested 3 hours after training (* P=0.019 *** P=0.0003). When re-tested at 24 hours, the response to the CS- decreases (*** P=0.0004 **P=0.004 n=10). B. At 3 hours we observe that responses to the CS- are correlated to the CS+ but that correlation is not present at 24 hours, reflection of the decrease in CS- responses. C. Memory association between events separated by 30 minutes is still observed if animals are tested 72 hours after training (ANOVA F=23.38 P<0.0001; ****P<0.0001 ***P=0.0002 n=10). D. Animals tested at 72 hours post-training still show a correlation between CS- and CS+ fear responses. Error bars represent SEM.

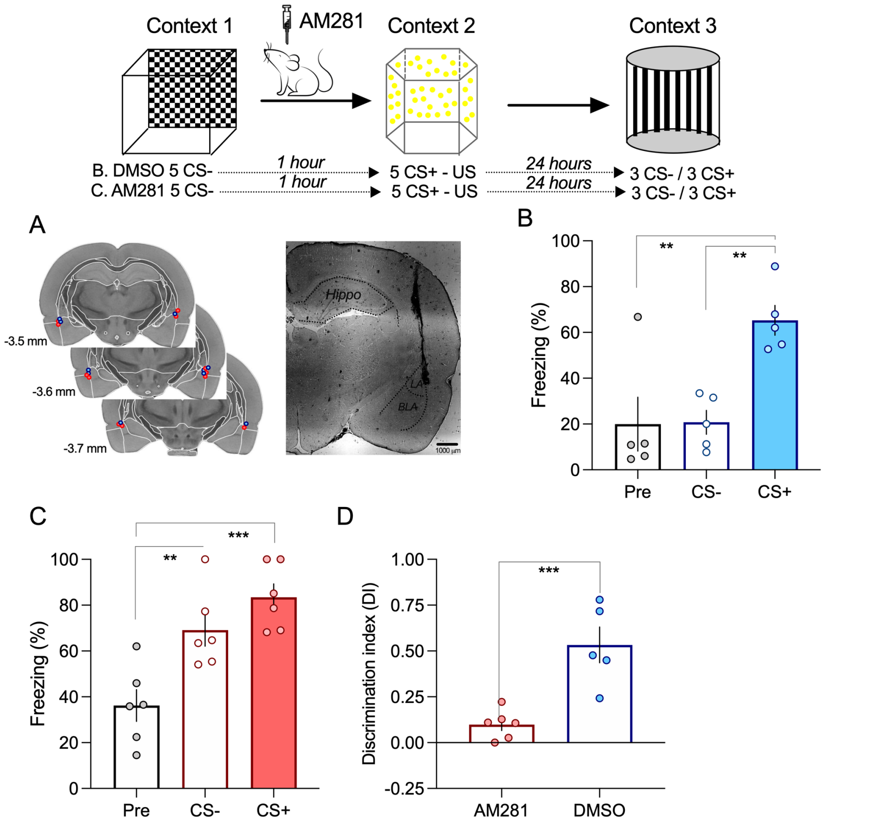

Supplementary Figure 3

Supplementary Figure 3. Inhibiting CB1 receptors by AM281 application in the lateral amygdala increases fear memory association. A. Placement of internal cannula. B. In control conditions, animals do not associate events separated by one hour (ANOVA F=9.7 P=0.0031; **P=0.006 **P=0.006 n=6). C. Inhibition of CB1R significantly increases fear responses for the neutral event (ANOVA F=13.47 P=0.0004; **P=0.008 ***P=0.0004 n=5). D. Discrimination is significantly decreased if CB1R are inhibited (Unpaired two-tailed t-Test **p=0.0013). Error bars represent SEM.

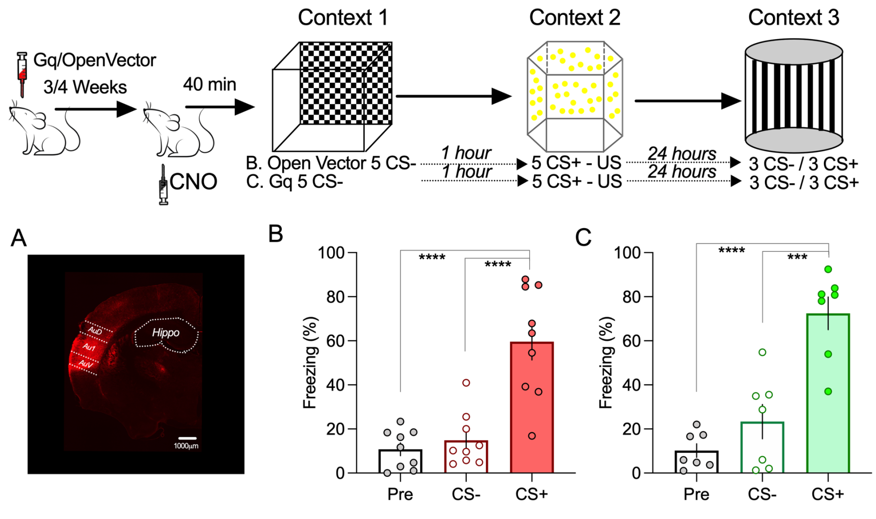

Supplementary Figure 4

Supplementary Figure 4. A. Injection in the primary auditory cortex. B/C. No differences were observed between animals where the cortical input activity is increased by CNO Gq activation versus animals expressing an open vector (B. Gq ANOVA F=23.52 P<0.0001; ****P<0.0001 ****P<0.0001 n=9; C. Open Vector ANOVA F=25.83 P<0.0001; ****P<0.0001 ***P=0.0001 n=7). Error bars represent SEM.

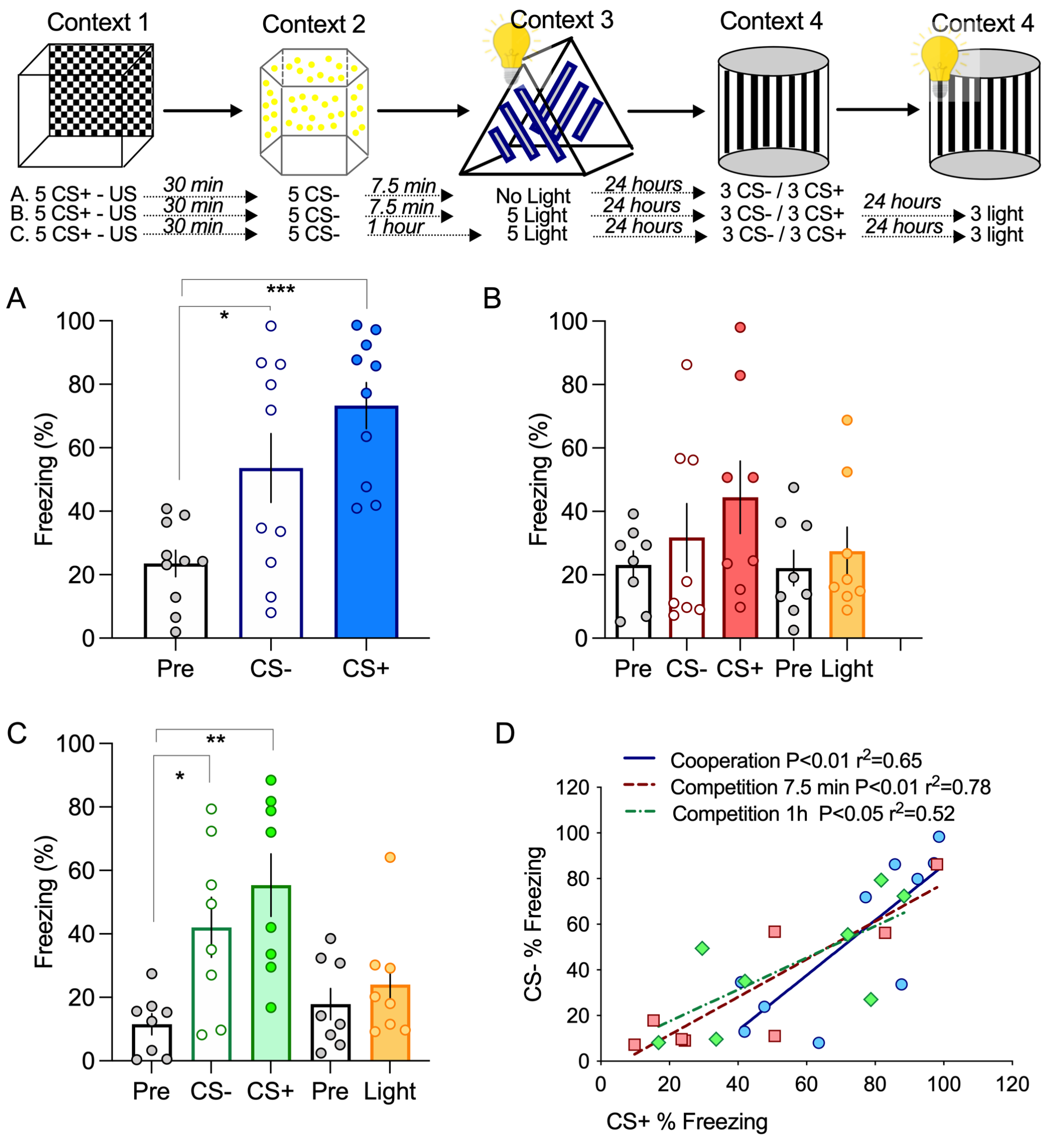

Supplementary Figure 5

Supplementary Figure 5. Exposing animals to a third event leads to competition. A. Events are associated by cooperation with a 30 min interval (ANOVA F=10.04 P=0.0005; *P=0.031 **P=0.0004 n=10). B. If animals are exposed to a third event, consisting of a different context paired with a light stimulus within 7.5 min, responses to the CS+ and CS- decrease and are no longer different from pre-freezing levels (ANOVA F=1.3 P=0.29 n=8). C. Increasing the time interval between events 2 and 3 to 1 hour prevents competition (ANOVA F=7.67 P=0.0031; *P=0.037 **P=0.0027; Unpaired two-tailed t-Test pre-freezing vs Light p=0.46 n=8) D. Although the third event induces competition, the responses to the CS+ and CS- are still linked. All conditions show a positive correlation, indicating the strong link between events 1 and 2. Error bars represent SEM.

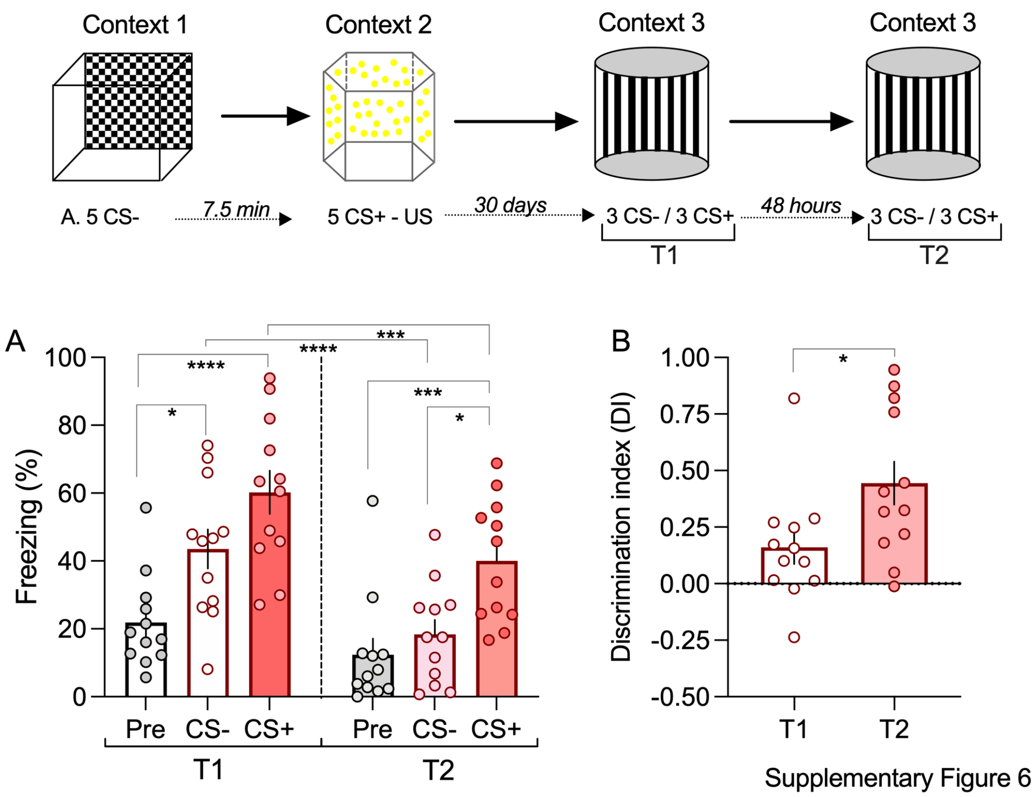

Supplementary Figure 6. Memory association is enduring. A. Animals show an intact memory association even if tested one month after training. As before, reactivation of memory led to a significant decrease in the response to the neutral (CS-) event (Two-way repeated measures ANOVA F=51.36 P<0.0001; Time 1 *P=0.01 ****P<0.0001 Time 2 ****P<0.0001 ***P=0.0002 n=12; Time 1 – Time 2 CS+ *** P=0.002 CS- P<0.0001). B. Memory reactivation significantly increased discrimination between the fearful and neutral event, by selectively decreasing the fearful response to the neutral event (Unpaired two-tailed t-Test *p=0.027). Error bars represent SEM.

Supplementary Statistical Table 1 - Madeira et al

Figure	Group	N	Statistical Test		Effect Size (eta squared)	Tukey Multiple comparisons test		P value
Figure 1	Cooperation 7.5 min	n=9	One Way ANOVA	F(2,24)=20.87 P<0.0001	0.6349	Pre-freezing vs. CS-	***	0,0007
						Pre-freezing vs. CS+	****	<0.0001
						CS- vs. CS+	ns	0,1346
	Cooperaion 30 min	n=9	One Way ANOVA	F(2,24)=8.98 P=0.0012	0.4281	Pre-freezing vs. CS-	*	0,0268
						Pre-freezing vs. CS+	***	0,001
						CS- vs. CS+	ns	0,3695
	Cooperation 1h	n=10	One Way ANOVA	F(2,27)=53.47 P<0.0001	0.7984	Pre-freezing vs. CS-	ns	0,9845
						Pre-freezing vs. CS+	****	<0.0001
						CS- vs. CS+	****	<0.0001
	Discrimination Index		One Way ANOVA	F(2,25)=20.13 P<0.0001	0.6169	7.5min vs. 30min	ns	0,92
						7.5min vs. 1h	****	<0.0001
						30min vs. 1h	****	<0.0001
	Cooperation 7.5 min		Linear Regression	F=0.2437	R square=0.03364		ns	p=0.6367
	Cooperation 30 min		Linear Regression	F=24.58	R square=0.7783		***	p=0.0016
	Cooperation 1h		Linear Regression	F=0.0763	R square=0.0094		ns	p=0.789
Figure 2	Thalamic Cooperation 1h Open Vector	n=8	One Way ANOVA	F(2,21)=33.73 P<0.0001	0.7626	Pre vs. CS-	ns	0,3147
						Pre vs. CS+	****	<0.0001
						CS- vs. CS+	****	<0.0001
	Thalamic Cooperation 1h Gq	n=10	One Way ANOVA	F(2,27)=7.883 P=0.002	0.3687	Pre vs. CS-	*	0,0363
						Pre vs. CS+	**	0,0017
						CS- vs. CS+	ns	0,4259
	Thalamic Cooperation 1h Gq no CS-	n=8	One Way ANOVA	F(2,21)=25.74 P<0.0001	0.7102	Pre vs. CS-	ns	0,9164
						Pre vs. CS+	****	<0.0001
						CS- vs. CS+	****	<0.0001
	Thalamic Cooperation 1h Gq saline	n=10	One Way ANOVA	F (2, 27) = 86,45 P<0,0001	0,8649	Pre vs. CS-	ns	0,6021
						Pre vs. CS+	****	<0,0001
						CS- vs. CS+	****	<0,0001
	Discrimination Index		One Way ANOVA	F(2,32)=12.71 P<0.0001	0.5438	OpenVector vs. Gq	*	0,0296
						OpenVector vs. Gq No CS-	ns	0,209
						OpenVector vs. Gq_saline	ns	0,1324
						Gq vs. Gq No CS-	****	<0,0001
						Gq vs. Gq_saline	****	<0,0001
						Gq No CS- vs. Gq_saline	ns	0,999
						Gq vs. Gq No CS-	****	<0.0001
	Cooperation 1h Open Vector		Linear Regression	F=0.0365	R square=0.006		ns	p=0.85
	Cooperaton 1h Gq		Linear Regression	F=60.38	R square=0.883		****	p<0.0001
	Cooperation 1h Gq no CS-		Linear Regression	F=2.15	R square=0.26		ns	p=0.19
	Cooperation 1h Gq saline		Linear Regression	F=0.33	R square=0,039		ns	p=0,5830
Figure 3	Competition 7.5 no Light	n=9	One Way ANOVA	F(2,24)=8.793 P=0.0014	0.4229	Pre vs. CS-	*	0,012
						Pre vs. CS+	**	0,0016
						CS- vs. CS+	ns	0,6801
	Competiton 7.5 min	n=8	One Way ANOVA	F(2,21)=16.02 P<0.0001	0.6041	Pre vs. CS-	ns	0,4964
						Pre vs. CS+	****	<0,0001
						CS- vs. CS+	**	0.0011
			Unpaired two-tailed t-Test	Pre vs Light	0.02993	t=0.6572, df=14	ns	0,5217
	Competition 7.5 no Light		Linear Regression	F=5.691	R square=0.44		*	p=0.0485
	Competiton 7.5 min		Linear Regression	F=0.1571	R square=0.0255		ns	p=0.7056
	Competition 7.5 min no CS-	n=6	Unpaired two-tailed t-Test	Pre vs CS+	0.5004	t=3.165, df=10	*	0,01
			Unpaired two-tailed t-Test	Pre vs Light	0.062	t=0.8186, df=10	ns	0,4321
Figure 4	Competition Open Vector	n=7	One Way Anova	F(2,18)=9.001 P=0.002	0.5000	Pre vs. CS-	*	0,0352
						Pre vs. CS+	**	0,0016
						CS- vs. CS+	ns	0,3368
	Competition ChR2	n=9	One Way Anova	F(2,24)=34.17 P<0.0001	0.7401	Pre vs. CS-	ns	0,7523
						Pre vs. CS+	****	<0.0001
						CS- vs. CS+	****	<0.0001
	Discrimination Index		Unpaired two-tailed t-Test	ChR2 vs Open Vector	0.4516	t=3.395, df=14	**	0,0044
	Competition Open Vector		Linear Regression	F=12.51	R square=0.7144		*	p=0.0166
	Competiton ChR2		Linear Regression	F=0.1104	R square=0.0155		ns	p=0.7494
Figure 5	Cooperation 7.5 min Context Only	n=12	One Way ANOVA	T2 F(2,33)=15.86 P<0.0001	0.4901	Pre vs. Cs-	***	0,0005
						Pre vs. Cs+	****	<0.0001
						Cs- vs. Cs+	ns	0,4938
	Cooperation 7.5 min Reactivation CS-/CS+	n=11	Two-way Repeated Measures ANOVA	Time<br>Column	F(1,30)= 7.969 P=0.0084<br>F(2,30)=15.42 P<0.0001	Time 1		
						Pre vs. CS-	**	0,0072
						Pre vs. CS+	***	0,0002
						CS- vs. CS+	ns	0,489
						Time 2		
						Pre vs. CS-	ns	0,8906
						Pre vs. CS+	***	0,0001
						CS- vs. CS+	***	0,0006
						Time 1 - Time 2		
						Pre	ns	0,89
						CS-	**	0.0024
						CS+	ns	0.99
	Cooperation 7.5 min Reactivation CS+ only	n=9	T1 Unpaired two-tailed t-Test	Pre vs CS+	0.4797	t=3.841, df=16	**	0,0014
			T2 One Way ANOVA	F(2,24)=10.06 P=0.0007	0.4560	Pre vs. CS-	ns	0,8034
						Pre vs. CS+	***	0,001
						CS- vs. CS+	**	0,0047
	Cooperation 7.5 min Reactivation CS- only	n=10	T1 Unpaired two-tailed t-Test	Pre vs CS-	0.2883	t=2.700, df=18	*	0,01
			T2 One Way ANOVA	F(2,27)=19.73 P<0.0001	0.5938	Pre vs. CS-	ns	0,4035
						Pre vs. CS+	****	<0.0001
						CS- vs. CS+	***	0,0002
	Discrimination index		One Way ANOVA	F(3,38)=6.539 P=0.0011	0.3505	Context vs CS+/CS-	*	0.0477
						Context Vs CS+	**	0.0011
						Context vs CS-	*	0.0122
						CS+/CS- vs CS+	ns	0.4412
						CS+/CS- vs CS-	ns	0.9303
						CS+ vs CS-	ns	0.8029

Supplementary Figure 1	Cooperation 7.5 min no CS-	n=8	One Way ANOVA	F(2,21)=22.64 P<0.0001	0,6832	Pre-freezing vs. CS-	ns	0,2166
						Pre-freezing vs. CS+	****	<0.0001
						CS- vs. CS+	***	0,0003
	Cooperation 7.5 min no CS+	n=8	One Way Anova	F (2, 21) = 0,8621 P=0.436	0,075	Pre-freezing vs. CS-	ns	0,2166
						Pre-freezing vs. CS+	****	<0.0001
						CS- vs. CS+	***	0,0003
			Unpaired two-tailed t-Test	Pre vs Context	0,27			
	Cooperation 7.5 min conterbalanced auditory cues	n=8	One Way Anova	F (2, 21) = 31,57 P<0.0001	0,7504	Pre-freezing vs. CS-	****	<0,0001
						Pre-freezing vs. CS+	****	<0,0001
						CS- vs. CS+	*	0,0462
Supplementary Figure 2	Cooperation 30 min test 3h	n=10	Two-way Repeated Measures ANOVA	Time<br>Column	F(1,27)= 1.89 P=0.18<br>F(2,27)=11.44 P<0.0001	Time 1		
						Pre vs. CS-	*	0,019
						Pre vs. CS+	***	0,0003
						CS- vs. CS+	ns	0,3565
						Time 2		
						Pre vs. CS-	ns	0,7035
						Pre vs. CS+	***	0,0004
						CS- vs. CS+	**	0,0049
			Linear Regression 3h	F=24.29	R square=0.752		**	0,0012
			Linear Regression 24h	F=4,07	R square=0.337		ns	0.07
	Cooperation 30 min test 72h	n=10	One Way Anova	F(2,27) =23,38 P<0.0001	0,634	Pre vs. CS-	***	0,0002
						Pre vs. CS+	****	<0,0001
						CS- vs. CS+	ns	0,1206
			Linear Regression	F=6,94	R square = 0,4646		*	0,029
Supplementary Figure 3	Cooperation 1h Control	n=5	One way ANOVA	F(2,12)=9.7 P=0.0031	0,6179	Pre-freezing vs. CS-	ns	0,9975
						Pre-freezing vs. CS+	**	0,0061
						CS- vs. CS+	**	0,0068
	Cooperation 1h AM281	n=6	One way ANOVA	F(2,15)=13.47 P=0.0004	0,6424	Pre-freezing vs. CS-	**	0,0081
						Pre-freezing vs. CS+	***	0,0004
						CS- vs. CS+	ns	0,3035
	Discrimination Index		Unpaired two-tailed t-Test	AM281 vs Control	0,6989	t=4.570, df=9	**	0.0013
Supplementary Figure 4	Cortical Cooperation 1h Open Vector	n=7	One Way ANOVA	F(2,18)=25.83 P<0.0001	0.7416	Pre vs. CS-	ns	0,3421
						Pre vs. CS+	****	<0.0001
						CS- vs. CS+	***	0,0001
	Cortical Cooperation 1h Gq	n=9	One Way ANOVA	F(2,24)=23.52 P<0.0001	0.6621	Pre vs. CS-	ns	0,8633
						Pre vs. CS+	****	<0.0001
						CS- vs. CS+	****	<0.0001
Supplemetary Figure 5	Cooperation 30 min	n=10	One Way ANOVA	F(2,27)=10.04 P=0.0005	0,4266	Pre vs. CS-	*	0,0313
						Pre vs. CS+	***	0,0004
						CS- vs. CS+	ns	0,2029
	Competition 7.5 min	n=8	One Way ANOVA	F(2,21)=1.302 P=0.293	0,1103	Pre vs. CS-	ns	0,7958
						Pre vs. CS+	ns	0,2662
						CS- vs. CS+	ns	0,6118
			Unpaired two-tailed t-Test	Pre vs Light	0,02194	t=0.5604, df=14	ns	0,5841
	Competition 1h	n=8	One Way ANOVA	F(2,21)=7.67 P=0.0031	0,4222	Pre vs. CS-	*	0,0374
						Pre vs. CS+	**	0,0027
						CS- vs. CS+	ns	0,4893
			Unpaired two-tailed t-Test	Pre vs Light	0,03948	t=0.7586, df=14	ns	0,4607
	Cooperation 30 min		Linear Regression	F=14.95	R square=0.65		**	p=0.0048
	Competition 7.5		Linear Regression	F=21.48	R square=0.78		**	p=0.0036
	Competition 1h		Linear Regression	F=6.59	R square=0.52		*	p=0.0425
Supplementary Figure 6	Cooperation 7.5 min Remote	n=12	Two-way Repeated Measures ANOVA	Time<br>Column	F(1,33)=51.36 P<0.0001<br>F(2,33)=12,90 P<0.0001	Time 1		
						Pre vs. CS-	*	0,0106
						Pre vs. CS+	****	<0.0001
						CS- vs. CS+	ns	0,0614
						Time 2		
						Pre vs. CS-	ns	0,6843
						Pre vs. CS+	***	0,0009
						CS- vs. CS+	*	0,0109
						Time1 - Time 2		
						Pre	ns	0,113
						CS-	****	<0.0001
						CS+	***	0,0002
	Discrimination Index		Unpaired two-tailed t-Test	T1 vs T2	0,2026	t=2.364, df=22	*	0.0273

Injections areas_all animals

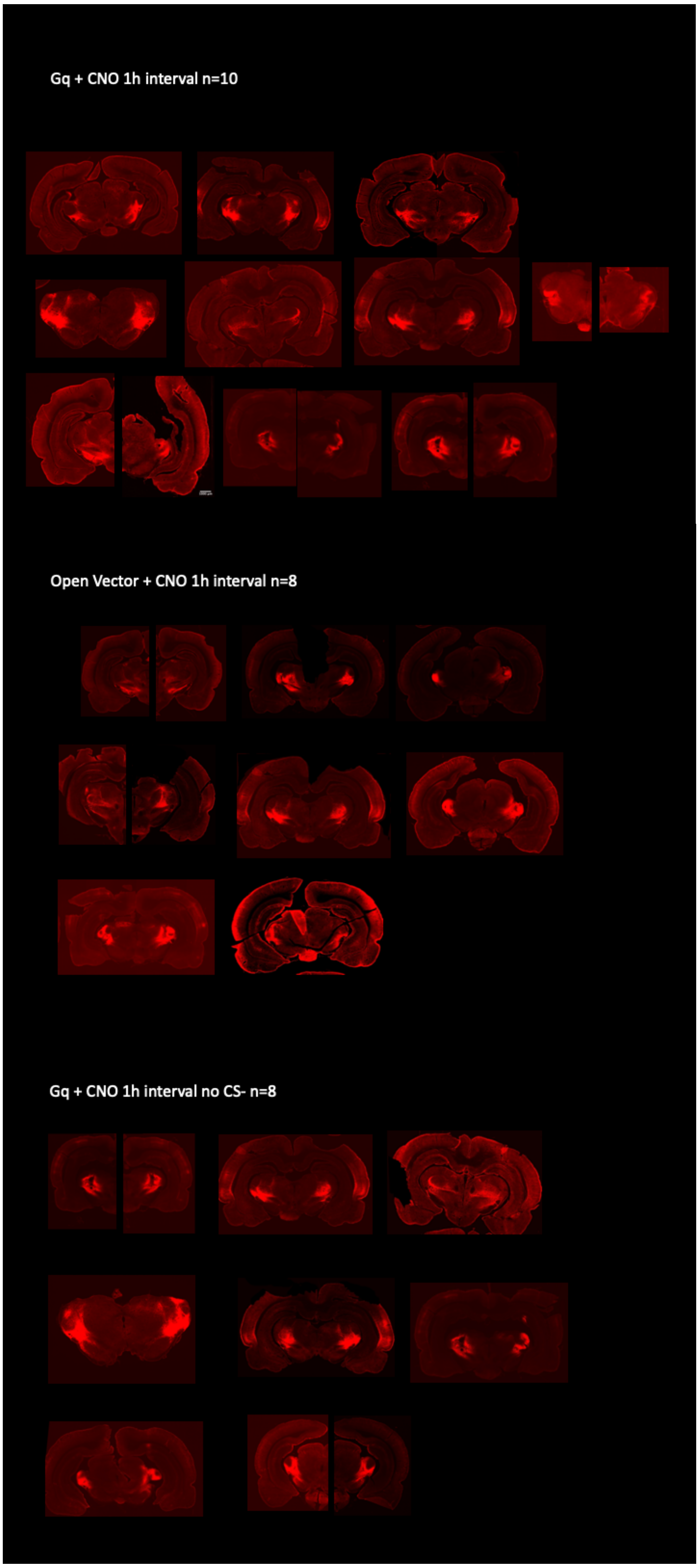
Gq + CNO 1h interval n=10
Open Vector + CNO 1h interval n=8
Gq + CNO 1h interval no CS- n=8

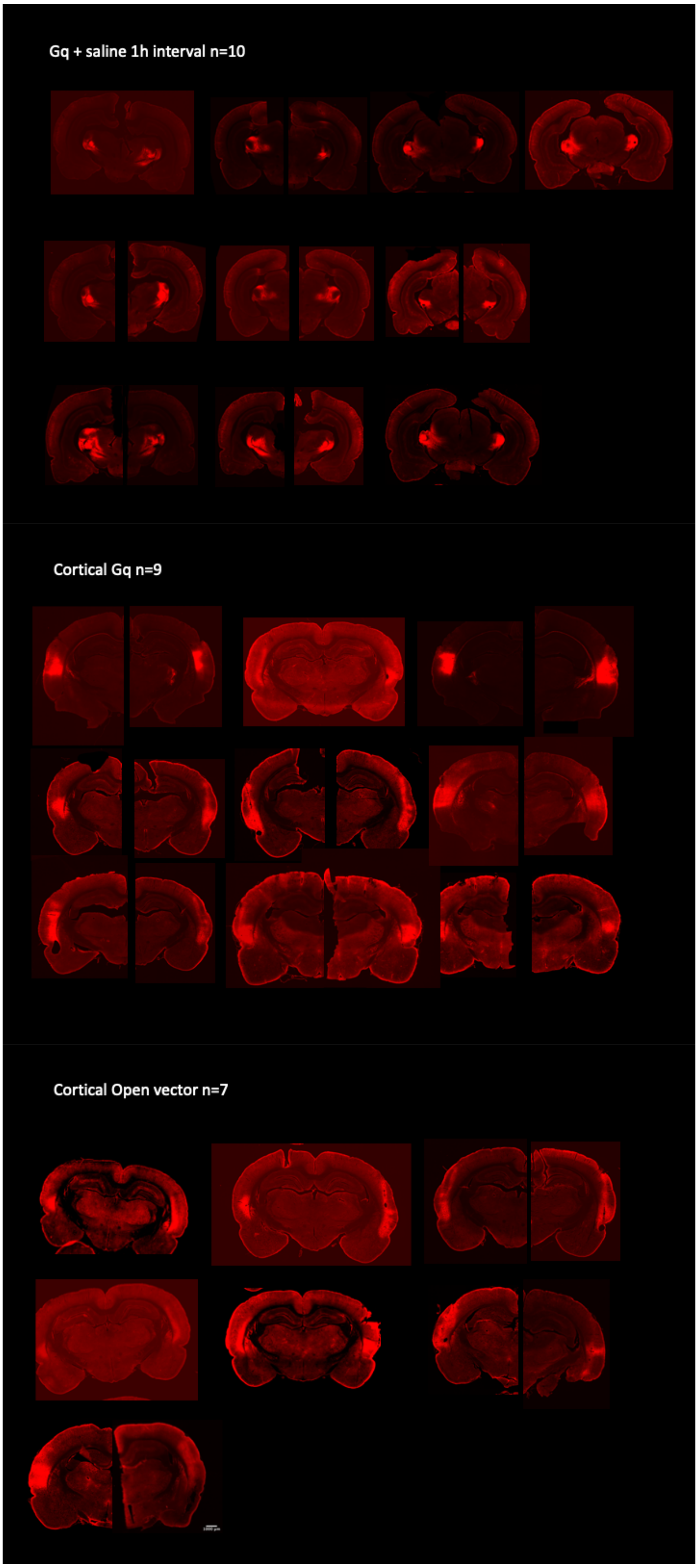
Gq + saline 1h interval n=10
Cortical Gq n=9
Cortical Open vector n=7

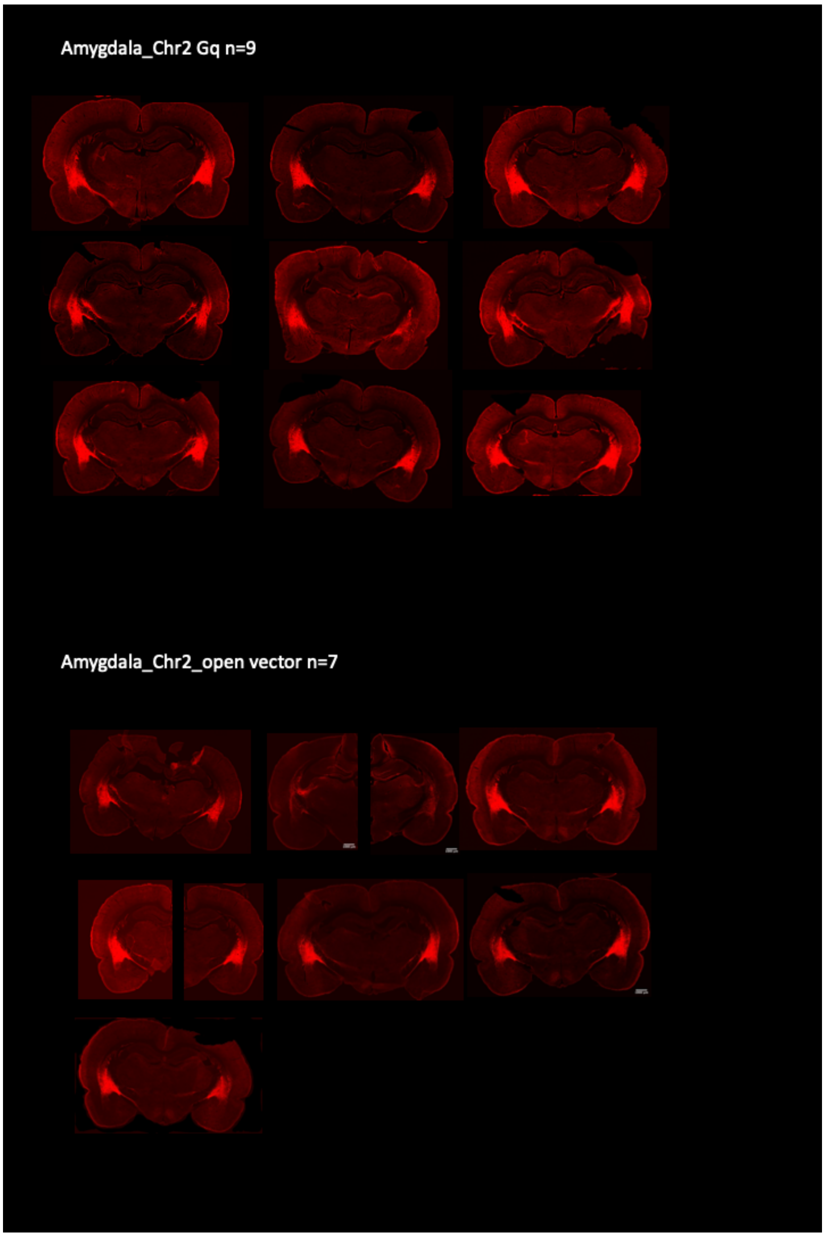

Conditioning Boxes

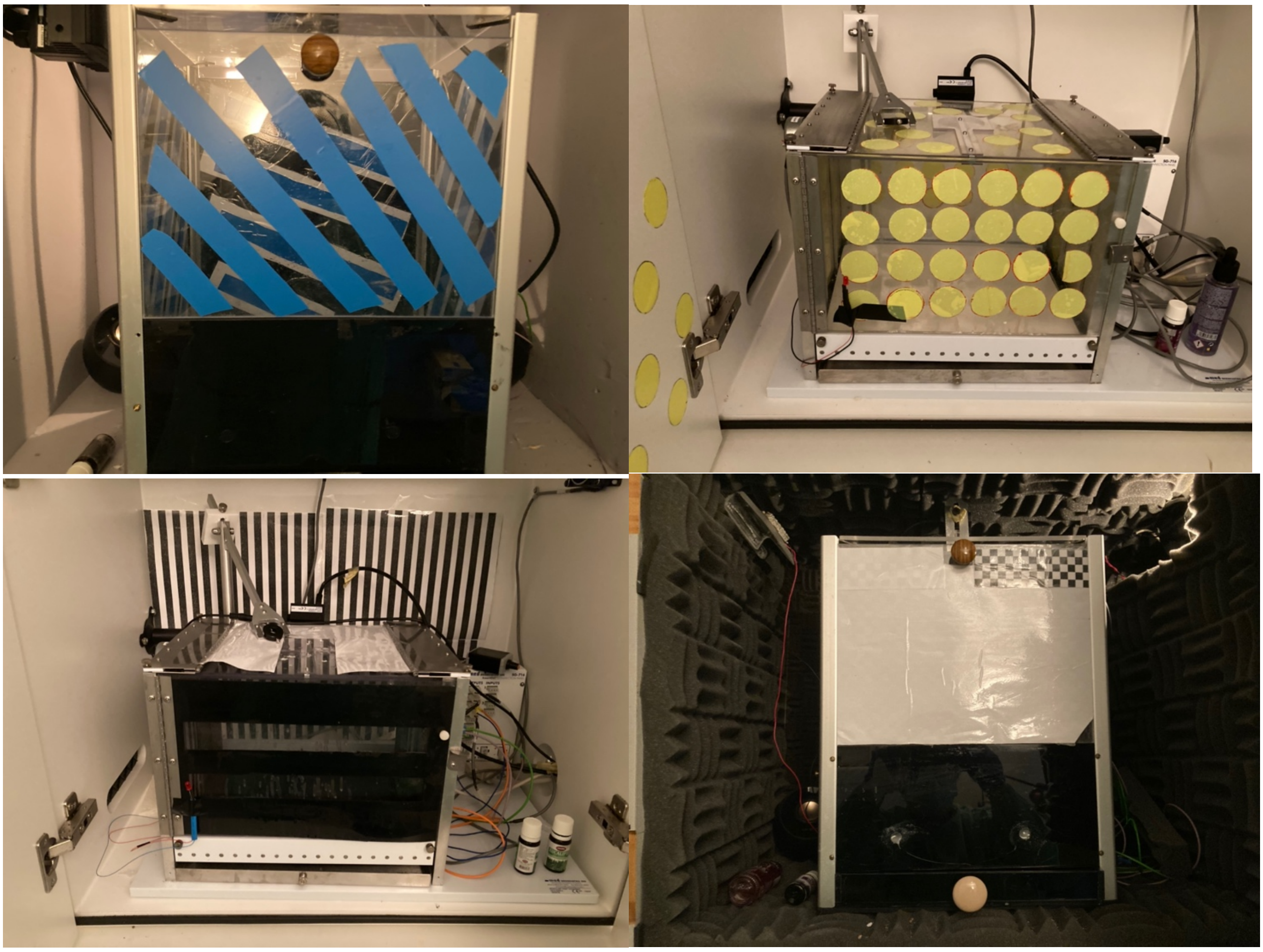

Supplement: Supplementary file 2 — Supplementary Information [file 42003_2025_8289_MOESM2_ESM.pdf]
