# Supplementary material for: Thalamic input to the lateral amygdala determines the temporal window of fear-memory association
Source: Commun Biol. 2025 Jun 5;8:873. doi: 10.1038/s42003-025-08289-0 (PMC12141508; doi:10.1038/s42003-025-08289-0)
Supplement: Supplementary file 3 — Description of Additional Supplementary Files [file 42003_2025_8289_MOESM3_ESM.pdf]

Description of Additional Supplementary Files

File name: Supplementary Data 1
Description: Source data behind graphs in the manuscript including all supplementary figures.
