# Supplementary material for: Thalamic input to the lateral amygdala determines the temporal window of fear-memory association
Source: Commun Biol. 2025 Jun 5;8:873. doi: 10.1038/s42003-025-08289-0 (PMC12141508; doi:10.1038/s42003-025-08289-0)
Supplement: Supplementary file 5 — Reporting Summary [file 42003_2025_8289_MOESM5_ESM.pdf]

nature portfolio

Corresponding author(s): Rosalina Fonseca
Last updated by author(s): May 15, 2025

Reporting Summary

Nature Portfolio wishes to improve the reproducibility of the work that we publish. This form provides structure for consistency and transparency in reporting. For further information on Nature Portfolio policies, see our Editorial Policies and the Editorial Policy Checklist.

Statistics

For all statistical analyses, confirm that the following items are present in the figure legend, table legend, main text, or Methods section.

n/a	Confirmed	
☐	☒	The exact sample size (*n*) for each experimental group/condition, given as a discrete number and unit of measurement
☐	☒	A statement on whether measurements were taken from distinct samples or whether the same sample was measured repeatedly
☐	☒	The statistical test(s) used AND whether they are one- or two-sided<br>*Only common tests should be described solely by name; describe more complex techniques in the Methods section.*
☐	☒	A description of all covariates tested
☐	☒	A description of any assumptions or corrections, such as tests of normality and adjustment for multiple comparisons
☐	☒	A full description of the statistical parameters including central tendency (e.g. means) or other basic estimates (e.g. regression coefficient) AND variation (e.g. standard deviation) or associated estimates of uncertainty (e.g. confidence intervals)
☐	☒	For null hypothesis testing, the test statistic (e.g. *F*, *t*, *r*) with confidence intervals, effect sizes, degrees of freedom and *P* value noted<br>*Give P values as exact values whenever suitable.*
☒	☐	For Bayesian analysis, information on the choice of priors and Markov chain Monte Carlo settings
☒	☐	For hierarchical and complex designs, identification of the appropriate level for tests and full reporting of outcomes
☐	☒	Estimates of effect sizes (e.g. Cohen's *d*, Pearson's *r*), indicating how they were calculated

Our web collection on statistics for biologists contains articles on many of the points above.

Software and code

Policy information about availability of computer code

Data collection	Behaviour videos were collected with Bonsai video capture https://bonsai-rx.org/.
Data analysis	All behaviour data was analysed with Bonsai https://bonsai-rx.org/. Graphical display of data and statistical analysis was done with Prism 9.

For manuscripts utilizing custom algorithms or software that are central to the research but not yet described in published literature, software must be made available to editors and reviewers. We strongly encourage code deposition in a community repository (e.g. GitHub). See the Nature Portfolio guidelines for submitting code & software for further information.

Data

Policy information about availability of data

All manuscripts must include a data availability statement. This statement should provide the following information, where applicable:

- Accession codes, unique identifiers, or web links for publicly available datasets
- A description of any restrictions on data availability
- For clinical datasets or third party data, please ensure that the statement adheres to our policy

All data is available upon request to the corresponding author. Numerical data is available as supplementary data and available at doi:10.5281/zenodo.15412058

Research involving human participants, their data, or biological material

Policy information about studies with human participants or human data. See also policy information about sex, gender (identity/presentation), and sexual orientation and race, ethnicity and racism.

Reporting on sex and gender	N/A
Reporting on race, ethnicity, or other socially relevant groupings	N/A
Population characteristics	N/A
Recruitment	N/A
Ethics oversight	N/A

Note that full information on the approval of the study protocol must also be provided in the manuscript.

Field-specific reporting

Please select the one below that is the best fit for your research. If you are not sure, read the appropriate sections before making your selection.

☒ Life sciences ☐ Behavioural & social sciences ☐ Ecological, evolutionary & environmental sciences

For a reference copy of the document with all sections, see nature.com/documents/nr-reporting-summary-flat.pdf

Life sciences study design

All studies must disclose on these points even when the disclosure is negative.

Sample size	The sample size was estimated based on existing published research and adjusted based on preliminary experiments.
Data exclusions	We excluded animals that displayed a pre-freezing behaviour, above the average+SD of the group at the test trial. This is clearly stated in the methods section. All data from animals with incorrect viral injections or cannula/optic fiber positions were excluded from data analysis.
Replication	We included detailed methods in the manuscript to ensure that experimental findings could be easily reproduced. All behavioral experiments were repeated with the number of animals specified in the manuscript. In some experimental conditions, experiments were replicated successfully due to control cohorts.
Randomization	Animals were randomly allocated to experimental groups. All experimental contexts were counterbalanced.
Blinding	Due to the training procedure, researchers cannot be blind to the specific protocol in the behavioral and optogenetic experiments. A researcher blinded to the experimental groups confirmed the data analysis.

Reporting for specific materials, systems and methods

We require information from authors about some types of materials, experimental systems and methods used in many studies. Here, indicate whether each material, system or method listed is relevant to your study. If you are not sure if a list item applies to your research, read the appropriate section before selecting a response.

Materials & experimental systems

n/a	Involved in the study	
☒	☐	Antibodies
☒	☐	Eukaryotic cell lines
☒	☐	Palaeontology and archaeology
☐	☒	Animals and other organisms
☒	☐	Clinical data
☒	☐	Dual use research of concern
☒	☐	Plants

Methods

n/a	Involved in the study	
☒	☐	ChIP-seq
☒	☐	Flow cytometry
☒	☐	MRI-based neuroimaging

Animals and other research organisms

Policy information about studies involving animals; ARRIVE guidelines recommended for reporting animal research, and Sex and Gender in Research

Laboratory animals	In this study, we use wild-type Sprague-Dawley rats.
Wild animals	N/A
Reporting on sex	All animals used in this study are males, as stated in the methods section.
Field-collected samples	N/A
Ethics oversight	All procedures in animals were approved by the Portuguese Veterinary Office (Direcção Geral de Veterinária e Alimentação — DGAV) and the ethics committee of i3S. This is stated in the methods section.

Note that full information on the approval of the study protocol must also be provided in the manuscript.

Plants

Seed stocks	N/A
Novel plant genotypes	N/A
Authentication	N/A
